# The role of nutrients in human neurodevelopment and their potential to prevent neurodevelopmental adversity

**DOI:** 10.3389/fnut.2022.992120

**Published:** 2022-11-22

**Authors:** Sarah Heland, Neville Fields, Stacey Joan Ellery, Michael Fahey, Kirsten Rebecca Palmer

**Affiliations:** ^1^Monash Women’s and Newborn, Monash Health, Clayton, VIC, Australia; ^2^Department of Obstetrics and Gynaecology, Monash University, Clayton, VIC, Australia; ^3^The Ritchie Centre, Hudson Institute of Medical Research, Clayton, VIC, Australia; ^4^Paediatric Neurology Unit, Monash Children’s Hospital, Clayton, VIC, Australia; ^5^Department of Paediatrics, Monash University, Clayton, VIC, Australia

**Keywords:** nutrition, neurodevelopment, neurodevelopment adversity, nutraceuticals, prenatal nutrition, pregnancy, fetal development

## Abstract

Nutritional deficits or excesses affect a huge proportion of pregnant women worldwide. Maternal nutrition has a significant influence on the fetal environment and can dramatically impact fetal brain development. This paper reviews current nutritional supplements that can be used to optimise fetal neurodevelopment and prevent neurodevelopmental morbidities, including folate, iodine, vitamin B12, iron, and vitamin D. Interestingly, while correcting nutritional deficits can prevent neurodevelopmental adversity, overcorrecting them can in some cases be detrimental, so care needs to be taken when recommending supplementation in pregnancy. The potential benefits of using nutrition to prevent neurodiversity is shown by promising nutraceuticals, sulforaphane and creatine, both currently under investigation. They have the potential to promote improved neurodevelopmental outcomes through mitigation of pathological processes, including hypoxia, inflammation, and oxidative stress. Neurodevelopment is a complex process and whilst the role of micronutrients and macronutrients on the developing fetal brain is not completely understood, this review highlights the key findings thus far.

## Introduction

Neurodevelopment is, unsurprisingly, extremely complex and involves multiple processes including neurulation, neuronal proliferation and migration, apoptosis, synaptogenesis, and myelnation (1). It is a process that begins in the weeks following conception and continues through to adulthood, with genetics, epigenetics, and environment all influencing the outcome (1, 2). Normal human neurodevelopment also requires a tightly regulated balance of numerous reactive oxygen species (3, 4). Defects in these processes can lead to highly variable outcomes, ranging from mild to severe neurobehavioural morbidities, to mortality that may occur in fetal life, childhood, and beyond (5).

Optimal maternal nutrition is important not only for the health of the mother but also the offspring. It has been shown to have an impact not only on cognition, but also development of non-communicable diseases including type 2 diabetes and atopic conditions, such as atopic dermatitis (6, 7). Nutritional deficits affect a huge proportion of pregnant women worldwide, with some regions of the world having up to 40% of pregnant women underweight with a body mass index (BMI) <18.5 kg/m^2^ and 42% of pregnant women worldwide having iron deficiency anaemia (8). Maternal nutrition, before and during pregnancy, dramatically influences the fetal environment and can significantly impact fetal brain development (9). The leading role nutrients play in neurodevelopment was first highlighted in the 1960s, when folic acid (vitamin B9) was shown to assist both cellular proliferation and the successful closure of the neural tube, an essential structure for brain and spinal column development (10). Subsequently, other nutrients have been identified whose absence or deficit may also adversely affect neurodevelopment.

This review will detail the role of nutrients in supporting fetal neurodevelopment, exploring the importance of timing the delivery of nutrients in pregnancy and the promising new advances that, when used appropriately, might aid in preventing neurodevelopmental adversity.

As we will outline in this paper, several antenatal supplements/nutrients are used in pregnancy to correct maternal nutritional deficiencies, while others are used to promote improved neurodevelopmental outcomes through mitigation of pathological processes, including hypoxia, inflammation, and oxidative stress. The micronutrients explored within this review have current global recommendations for supplementation in pregnancy. While there is the potential that additional micronutrients, such as copper, zinc, and lutein, may afford benefits in neurodevelopment evidence is currently lacking to inform clinical use and as such they are not recommended as specific supplements in pregnancy. It should be noted, however, that the 2020 WHO (11, 12) nutritional interventions update on multiple micronutrient supplements (MMS) during pregnancy has included both zinc and copper in its recommendation, but only in the context of rigorous research. Of note, the currently available MMS United Nations International Multiple Micronutrient Antenatal Preparation (UNIMMAP) pregnancy multivitamin formula does include both copper (2 mg) and zinc (15 mg) formulated for use in micronutrient poor settings such as low–middle income countries (11). Lutein is still currently under investigation for its use in pregnancy, but does have promising initial findings that increased maternal intake improves childhood behaviour and verbal intelligence (13). Unsurprisingly the story is complex, and the role of micronutrients and macronutrients during pregnancy, and their effect on the developing fetus, are still far from fully understood.

## Current nutritional supplements to optimise fetal neurodevelopment

### Folate

Neural cells divide rapidly in the embryo, especially in the first trimester as the fetal brain forms (14). Primary neurulation, the folding of the neural plate to form the neural tube, occurs on days 21–28 of gestation. The successful closure of the neural tube is essential for subsequent brain and spinal cord development, with errors in this process leading to incomplete plate fusion (15). If this occurs in the cranial region, it can result in anencephaly, whereas caudal occurrence causes spina bifida (15).

A substantial breakthrough occurred in the 1960s with the identification that folate supplementation could reduce the risk of neural tube defects (NTD) (10). Folate is found in green leafy vegetables and yeast extract, while its more stable synthetic form, folic acid, can be found in breads, cereals, and supplements (16). Folate is involved in neural cell proliferation and differentiation, reducing apoptosis, and maintaining DNA synthesis (14). Folate’s importance in supporting fetal development is further highlighted, by the multiple mechanisms that exist to support folate transport across the placenta, including the folate receptor, the reduced folate carrier and the proton-coupled folate transporter (17). Consequently, folate requirements are 5–10 times higher in pregnant women compared with the non-pregnant population (18). As humans cannot synthesise folate *de novo*, this increased requirement must be achieved through increasing dietary intake or *via* supplementation (18).

Women planning a pregnancy should take 0.4–0.8 mg of folate at least 1 month preconception until the end of the first trimester (19, 20). Given its impact on early neural development, these recommendations highlight the importance of timing folate supplementation to optimise embryonic development. Practical delivery of this benefit is complicated because approximately 50% of pregnancies are unplanned (16), and one in three people globally are estimated to suffer some form of malnutrition (19). Consequently, many countries worldwide have fortified cereal grain products with folic acid, resulting in a 40–80% reduction in the prevalence of spina bifida and anencephaly, which now complicates approximately 11.7–21.9 per 10,000 births (5, 14, 16, 21).

While folate’s periconceptional and first trimester benefits have been well established, its role in the second and third trimesters is less well known. Studies have shown that continuing folic acid supplementation in the second and third trimester has resulted in changes in DNA methylation in the cord blood of genes related to brain development, with lower levels of methylation in LINE-1, IFG2, and BDNF genes (22). A randomised controlled trial is currently underway examining the effect of folate supplementation beyond the first trimester on maternal plasma unmetabolised folic acid (23). However, the long-term real-world implications of these findings are unknown, and further epigenome-wide studies are necessary to explore the potential impact of folate use beyond the first trimester. Interestingly, a study involving 45,300 children showed an association between folic acid and multivitamin supplements and a reduced risk of autism spectrum disorders in the offspring (24). These findings were consistent for both maternal exposure to folic acid or multivitamins either before pregnancy (RR 0.39; 95% CI 0.30–0.50; *P* < 0.001) or during pregnancy (RR 0.27, 95% CI 0.22–0.33; *P* < 0.001), however, this is an area that requires further research (24). Further research is demonstrating that folic acid use and levels in the second trimester positively correlate with fetal growth (25, 26). The ongoing research into the benefit of folate beyond the first trimester will be an area to watch in the future.

Furthermore, patients at higher risk for NTD are recommended to use high dose folate, 5 mg per day from 2 months pre-pregnancy until the end of the first trimester (27). This recommendation applies to patients with a previous pregnancy affected by NTDs, malabsorption disorders, obesity, diabetes, or on specific medications, such as anti-epileptics or folate antagonists (27).

However, there are concerns surrounding the potential risks of high-dose folate use. As folate supports rapidly dividing cells, there is a theoretical concern that it may promote cancer growth in susceptible women. Research shows that folate may have a disparate impact on different types of cancer, increasing the risk of breast cancer, but not colon cancer (28, 29). As folate is a cofactor for one-carbon transfers and involved in complex biological processes, it was postulated that it might play a role in epigenetic modifications resulting in increased rates of atopic disease in the offspring (30). This concern may be relevant given rising allergy rates (31). But the evidence on whether maternal supplementation with folic acid is associated with atopy, reactive airway diseases, and increased risk of wheeze in early childhood is conflicted and of variable quality (31, 32). There does however appear to be a “U-shaped” relationship between the development of autism spectrum disorders and increasing folic acid supplementation, with a study of 1,257 mother-infant pairs suggesting that both inadequate and excessive folic acid is detrimental to the fetus. This same study found a 2.5-times increased risk of autism spectrum disorders with high maternal plasma folate levels (>60.3 nmol/L) (33). A recent systematic review has highlighted the conflicting evidence of folic acid supplementation pre-pregnancy and perinatally with autism development, highlighting challenges of comparing studies with variable methylene tetrahydrofolate reductase (MTHFR) genotype reporting and limited length of follow-up periods for neurodevelopmental assessments (34).

Given these potential adverse effects of folic acid supplementation, women should ensure they are on the minimal dose possible to reduce the risk of NTDs while avoiding the potential risks. Existing guidelines should be followed; however, several areas would benefit from further research. These include determining the benefit of ongoing folic acid supplementation beyond the first trimester, whether folic acid and widespread food fortification may be associated with harm, and whether serum folate levels should be assessed pre-conception to guide the need for additional supplementation particularly those patients with complex genetic, medical, or surgical co-morbidities who currently would receive high dose folic acid supplementation (35). Finally, further research would be beneficial in determining the lowest effective dose in many subsets, such as if there can be a tapered dose of folate depending on a patient’s BMI.

### Iodine

Iodine is a trace element found in iodised salt, fish, and grains and is essential for the production of thyroid hormones, thyroxine (T4) and triiodothyronine (T3), and these hormones are critical for neurodevelopment (36, 37). During the first half of pregnancy, the fetus relies on the transplacental passage of thyroxine from the mother, which is essential for supporting the development of the fetal brain through neuronal migration and myelination (37). During pregnancy, maternal iodine requirements increase to support the production of maternal thyroid hormone needed to support maternal and fetal needs (38). If the mother is nutritionally unable to meet this increased demand, this can cause irreversible damage to the fetal brain (39).

Iodine deficiency is the leading global cause of preventable impaired mental function (38). It can cause neurodevelopmental issues, including motor function deficits, cognitive impairment, and behavioural disorders (37, 39). Resultantly, iodine supplementation in pregnancy is supported and it is recommended that women take 250 micrograms (mcg) daily throughout pregnancy (40).

A recent randomised control trial (RCT) demonstrated that iodine supplementation in pregnancy improved mild maternal iodine deficiency to adequate levels with a positive impact on maternal thyroglobulin (41). An extension of this study will explore the effect of maternal iodine supplementation on the offspring’s neuropsychological development; findings likely to inform the future use of iodine supplementation in areas of mild to moderate deficiency to optimise neurodevelopmental outcomes in children (Trial registration number: NCT02378246) (42).

While the benefit of iodine supplementation in areas of severe deficiency is well established, a systematic review and meta-analysis found that recommendations for iodine supplementation in areas with only mild to moderate deficiency are not supported by quality evidence (43, 44). This is important to consider, as evidence suggests maternal intake of 150μg/*day*, in pregnancy can detrimentally affect the psychomotor achievements of infants (45).

### Choline

Choline is a methyl donor nutrient that can be produced by the human liver or obtained from either animal products or plant foods, such as nuts, legumes, and cruciferous vegetables (14). It is a precursor for phospholipids, acetylcholine, and the methyl donor betaine (46). Choline is transferred across the placenta and choline concentrations in umbilical cord plasma were approximately three times those in maternal plasma (47). Choline plays a role in several aspects of fetal brain development including neural tube closure in humans, and hippocampal development in animals, however further study is needed to determine if this extends to humans (48, 49).

It is estimated that 90% of pregnant women in the USA do not meet the recommended intake of choline (50). However, studies of maternal supplementation with choline have had varying results. An RCT of mothers taking 930 versus 480 mg choline per day showed faster processing speeds in the infants of the 930 mg group (51). A study with maternal and infant choline supplementation from the second trimester through to the third month postpartum showed that supplementation may be associated with better sensory gating (52). Furthermore, maternal choline concentrations and dietary intake have been shown to have an inverse association with neural tube defects independent of folate (48, 53). Conversely, choline levels in the cord blood has been found to have no impact on IQ scores in children at 5 years or age (54). An RCT of women that took 750 mg/day of choline or a placebo from 18 weeks gestation to 90 days postpartum found no benefit on infant brain function (55). While further research is needed to determine prenatal choline’s long term impact on the brain, the current recommendation is that pregnant woman should aim for a choline intake of 450 mg/day (6). Importantly, many multivitamins do not contain choline and so it may need to be taken as an extra supplement (6).

### Vitamin B12

Vitamin B12, like vitamin B9 (folate), belongs to the B vitamin class of compounds; eight different chemically distinct, water soluble compounds, which sequester naturally together in meat, eggs and dairy products, despite being synthesised predominantly by plants (56). They are essential for normal cellular processes, particularly acting as cofactors for enzymatic reactions and in the synthesis and regulation of dopaminergic and serotonergic neurotransmitters (57). However, little clinical trial evidence currently exists for the other vitamin B compounds. Supplementation may be required in those with a vegetarian or vegan diet, or those suffering from gastrointestinal malabsorption (37, 58, 59). It is a cofactor for enzymes, methionine synthase, and L-methyl-malonyl-coenzyme A (59). These have a role in mitochondrial succinyl-CoA formation, cytosol methionine synthesis, and fat and protein metabolism. It is essential for neuronal structure and myelination (37, 60). Fetal levels of vitamin B12 are thought to be closely related to maternal levels, and transport across the placenta occurs bound to transcobalamin or haptocorrin (59). Infants born to mothers with adequate vitamin B12 levels have approximately 25 mcg stored at birth, but endogenous vitamin B12 stores in infants may be much lower in vitamin B12 deficient mothers (58).

There is evidence that low B12 levels are detrimental to fetal development but the data is inconsistent and confounded by comorbidities. A pooled analysis of case reports of infant vitamin B12 deficiency, all resulting from maternal deficiency (18 cases of pernicious anaemia, 28 cases of strict veganism, and 2 cases of “very low” maternal animal source food intake) where the infants were exclusively breastfed, found that 58% reported developmental delay and 32.5% had cerebral atrophy (58). Reassuringly, symptoms such as apathy, muscle hypotonia, anorexia, and involuntary movements of limbs and tongue can improve rapidly with infant supplementation (58). This was evident with approximately a third of infants with developmental delay born to B12 deficient mothers showing symptom resolution with treatment. However, 38% of offspring to mothers with pernicious anaemia and 50%, of those born to vegan mothers had persisting long-term developmental impairment, but it was noted that there was variability in the assessment of recovery, age, assays for plasma B12, and time after treatment (58). A randomised control trial in India found that healthy mothers supplemented with vitamin B12 had no significant impact on cognitive development in the offspring compared to placebo (61). However, multiple regression analysis showed that elevated maternal total homocysteine levels, adjusted for the treatment group, birthweight, parity, income, and home environment, were associated with poorer performance in the expressive language and fine motor domains of the Bayley Scales of Infant Development-III (61). As the amino acid, homocysteine, is metabolised by vitamin B12, raised levels may indicate vitamin B12 deficiency. However, high levels can also be due to other vitamin deficiencies, including folate, impacting the interpretation of these results.

In comparison, high maternal dietary intake of vitamin B12 in the second trimester of pregnancy shows a weak association with poor language development, as demonstrated by lower scores on the Peabody Picture Vocabulary Test III in 3-year-olds (62). Very high maternal plasma B12 levels (≥536.8 pmol/L) have also been associated with a 2.5-fold higher rate of autism spectrum disorder in offspring, suggesting that B12 supplementation should be carefully assessed rather than a blanket wide recommendation for treatment in pregnancy (33). Given these inconsistent findings, this area requires further long-term research to determine the impact that vitamin B12 deficiency can have on the cognitive outcome of offspring. Encouragingly there is an upcoming double blinded RCT that aims to assess the effect of vitamin B12 supplementation during pregnancy on infant neurodevelopmental outcomes (Trial Registration Number NCT04083560) (63).

### Iron

Iron is an essential element that both directly, through promoting neurogenesis and myelination, and indirectly, through the formation of haemoglobin and delivery of oxygen to developing tissues, has critical impacts on fetal neurodevelopment (64). It is the most common nutrient deficiency in the world (64). Fetal iron levels are dependent on the mothers’ iron stores, thereby increasing maternal iron requirements in pregnancy and increasing the risk of deficiency (37). After birth, infants cannot regulate the absorption of iron from the gut for the first 6–9 months, so good iron stores at birth are essential (64).

Iron deficiency can affect brain development. Deficiency alters gene expression affecting hippocampal development and function, including learning, memory, and cognition, and undergoes rapid neurogenesis in the late prenatal and the early postnatal period (64). Iron deficiency can also indirectly impact neurodevelopment by increasing the risk of low birth weight, which has been associated with delayed neurocognitive development (64).

There is evidence that the effects of fetal iron deficiency persist even with postnatal supplementation and correction. Children who were iron deficient at birth were studied at 5 years of age and scored lower than their iron sufficient counterparts on language, fine motor skills, and tractability (65). Therefore, it is essential to identify and correct iron deficiency in the prenatal period where possible (66).

Interestingly, high iron levels could potentially adversely impact neurodevelopment, with those in the highest quartile of iron levels having a lower full-scale intelligence quotient (65). Furthermore, daily oral iron supplementation (50 mg) in non-anaemic women could potentially be harmful with increased rates of small for gestational age neonates and hypertensive disorders (67). Therefore, while iron deficiency should be identified and corrected, it is important not to recommend routine iron supplements for every mother. This is supported by a study of 2,479 mother-child pairs assessed for maternal iron stores (serum ferritin) during early pregnancy, which found that those with the highest serum ferritin (mean = 170.3 microg/L) had children with lower child intelligence quotient scores and smaller brain volumes after exclusion of those with elevated C-reactive protein (CRP) levels (68). There are currently multiple trials evaluating iron replacement in pregnancy, and it will be beneficial to ensure follow-up of the neurodevelopmental outcomes in the offspring from these studies (69, 70).

### Vitamin D

Vitamin D, otherwise known as 25-hydroxyvitamin D, is a hormone that is endogenously produced in the skin by ultraviolet light exposure and activated through hydroxylation by the liver and kidneys or exogenously acquired through diet (71, 72). Fetal levels are dependent on maternal supply being actively transported across the placenta before being activated through hydroxylation by the fetal kidneys to 1,25-dihydroxyvitamin D, the active form of vitamin D (71, 73). Vitamin D contributes to neurodevelopment through multiple mechanisms, including neuronal differentiation, axonal connectivity, dopamine ontogeny, and transcription control of genes (71).

Studies have shown the varied impact of maternal vitamin D deficiency on fetal brain development. Tylavsky et al. (72) completed a study on 1,020 participants that found maternal vitamin D levels positively correlated with receptive language development, but not cognitive or expressive language, in offspring at 2 years of age. A Vietnamese prospective cohort study further demonstrated that low maternal vitamin D levels in late pregnancy were associated with reduced language development in offspring at 6-months of age (74). Both trials are consistent with a systematic review and meta-analysis of observational studies that showed lower scores on mental and language developmental tests in offspring born to mothers with vitamin D deficiency (75). While a contrasting study from Greece showed no association between maternal vitamin D concentrations and offspring cognitive function, it did suggest that normal vitamin D levels, in this case >50.7 nmol/l, may be protective in preschool children against behavioural difficulties such as attention deficit hyperactivity disorder (76). The impact of excessive maternal vitamin D supplementation remains unknown. While it has been linked to the development of fetal hypercalcaemia, no adverse neurodevelopmental outcomes have been reported (71). Current recommendations are for all pregnant women to take 400 IU of vitamin D daily throughout pregnancy (6, 77).

## Polyunsaturated fatty acids

Lipids, particularly phospholipids, are essential for the human brain, which is approximately 50% lipid content (78). Phospholipids are a diverse group of molecules with vital roles in most cell types within the brain, where they form membrane lipid bilayers, providing a functional barrier between the subcellular and surrounding environment (79). Polyunsaturated fatty acids (PUFAs) represent up to 35% of the total lipid content in the brain. The fetus is unable to manufacture PUFAs *de novo* in utero, which continues in early neonatal life, therefore normal neurodevelopment is dependent on adequate maternal dietary intake (80). The INCA2 survey found French pregnant and lactating women had inadequate PUFA intake of Omega-3 PUFAs ALA (four times lower than recommended daily intake) and docosahexaenoic acid (DHA) (10 times lower than recommended); however, the study did not report on neurodevelopmental outcomes (81). The Japan Environment and Children’s Study (JECS) assayed maternal fish and omega-3 PUFA intake and found lower dietary intakes were associated with reduced infant sleep duration at 1 year of age (82), which may be associated with adverse neurodevelopmental and neurobehavioural outcomes (83, 84). A large study of 1,553 Danish mother-child pairs found an association between lower mid-trimester plasma omega-3 PUFA levels and smaller brain volumes in children at ages 9–11 years (85).

Neurotoxicants, such as the highly lipophilic insecticide DDT (1,1,1-trichloro-2,2,-bis(p-chlorphenyl)ethane), have been shown to have detrimental effects on fetal neurodevelopment in retrospective cohort studies; an effect which was mitigated by increasing maternal intake of n-3 docosapentaenoic (DPA) fatty acid (80). This might in part be explained by the important role DPA serves as a precursor to resolvins and neuroprotections, with recent evidence of DPA both mitigating lipopolysaccharide (LPS) induced neuroinflammation and promoting M2 anti-inflammatory polarization of microglia (86). There is significant heterogeneity in outcomes of the previous studies investigating PUFAs in pregnancy, including DPA, eicosapentaenoic acid (EPA), and DHA, partially due to significant gene polymorphisms and the non-standardised testing of neurodevelopmental and neurobehavioural outcomes (87). For a comprehensive overview of PUFAs in brain development and neurodevelopmental disorders we recommend the review by Marinat et al. (87). A systematic review from 2013 including 11 randomised clinical trials with 5,272 participants found maternal supplementation with omega-3 PUFAs did not alter neurodevelopmental outcomes apart from two trials with a high risk of bias (88). Ultimately, maintaining the optimal level of PUFAs and aiming to optimise the omega-6: omega-3 ratio of PUFAs with selective omega-3 EPA monotherapy supplementation and limiting excess vegetable oils (reducing omega-6 PUFAs) typical in Western diets may result in improved neonatal neurodevelopmental outcomes (89), lower childhood body and abdominal fat (90), and improved maternal long-term cardiovascular outcomes (91). The International Federation of Gynecology and Obstetrics (FIGO) currently recommends increased requirements for PUFAs in pregnancy with Omega-6 PUFAs (13 g) and Omega-3 PUFAs (1.4 g) representing the two parent long-chain fatty acids linoleic acid (LA) and alpha-linolenic acid (ALA) vital for normal membrane lipid bilayer formation and hence fetal neurodevelopment (6).

## Promising nutraceuticals to protect fetal neurodevelopment

Normal fetal neurodevelopment requires adequate levels of many key nutrients as outlined. However, even with achieving optimal levels of these nutrients in pregnancy, pathological disorders of pregnancy can occur, such as preeclampsia, gestational diabetes, and fetal growth restriction. When pregnancy becomes complicated by these various pathological processes, the impact on placental functioning can consequently lead to reduced nutrient supply and fetal exposure to higher levels of pro-inflammatory mediators, oxidative stress, and hypoxia with resultant adverse neurodevelopmental outcomes (3, 92–95). A prime example of the impact of placental functioning on nutrient delivery and fetal exposure to pro-inflammatory mediators and reactive oxygen species is preeclampsia. Many preeclamptic animal models result from surgical manipulation of uterine blood supply, such as the Reduced Uterine Perfusion Pressure (RUPP) model commonly seen in rats (96). Whilst imperfect, this model does partly replicate the “Two Stage Model” of preeclampsia whereby reduced placental perfusion results in the clinical syndrome with fetal growth restriction, hypertension, and proteinuria (97). Placental morphology is altered in preeclamptic pregnancies with more oblong, thicker, and smaller surface area placentas compared to non-preeclamptic ones thought to contribute to impaired fetal nutrient delivery (98). These placental morphological changes in combination with maternal vascular malperfusion leading to reduced placental perfusion are the likely drivers behind impaired nutrient supply to the fetus (99). Reduced nutrient supply, oxidative stress, hypoxia, and inflammation are likely the key drivers in both the maternal and fetal injury seen in placental disorders and likely the major contributors to subsequent neurodevelopmental adversity (100, 101). These challenges can occur acutely, such as in birth asphyxia, or chronically, as in placental insufficiency, however with both having the potential for significant brain injury and adverse neurodevelopmental outcomes. There are a number of nutraceuticals currently under investigation that offer hope as potential therapies to minimise potential harm to the developing fetus and particularly to the developing brain (94, 102, 103).

### Sulforaphane

Sulforaphane is an isothiocyanate phytonutrient first discovered in the 1980s. It is found in cruciferous vegetables, such as broccoli, and has anti-inflammatory, anti-malignant, and antioxidant properties (104). Sulforaphane is a potent phase II detoxification enzyme inducer that works by activating nuclear factor erythroid 2-related factor 2 (Nrf2), which influences gene transcription through antioxidant response elements (AREs) (105). Unlike directly acting antioxidants, such as Vitamin C (ascorbic acid), which are consumed through their oxidative scavenging processes, sulforaphane can upregulate many different antioxidants through activating AREs situated in the promoter region of target genes (106). Nrf2 has been identified as a master regulator of cellular defenses to various stressors, including metabolic and oxidative stress, by regulating the expression of enzymes including heme oxygenase-1 (*HO-1*), thioredoxin (*TXN*), glucose 6-phosphate dehydrogenase (*G6PD*), and NAD(P)H:quinone oxidoreductase (*NQO1*) (106).

Many animal-based studies have shown potential benefits of sulforaphane in neurodevelopment due to its antioxidant and anti-inflammatory properties. Normal neurodevelopment of the fetus requires a tightly regulated balance of free radical formation and cellular antioxidant activity (3). However, when free radical formation and accumulation outstrips the ability of endogenous cellular antioxidant processes, oxidative stress occurs resulting in mitochondrial injury, impaired energy production, apoptotic cell death, and disordered neurodevelopmental pathways (3, 92, 107). A commonly encountered aromatic teratogen created from cooking meat at high heat, 2-amino-1-methyl-6-phenylimidazo[4.5-b]pyrimidine (PhIP) can cause embryonic death and neural tube defects, both of which were mitigated in chicken embryo culture containing sulforaphane (108).

Oxidative stress following acute brain inflammation, such as occurs with intrauterine fetal stroke, can result in the prolonged opening of the mitochondrial inner membrane permeability transition pore (PTP) leading to dysfunction of the electron transport chain and hence bioenergetic failure of neural cells (109). When bioenergetic failure occurs due to resultant ischaemia and hypoxia, this induces apoptosis and cell death dysregulating typical neurodevelopment (92). From this perspective, rat brain mitochondria when treated with sulforaphane under conditions of oxidative stress were able to resist PTP redox-regulated opening which may provide neuroprotective effects to the developing brain when exposed to oxidative stress (110). Similarly, cultured cortical neurons exposed to sulforaphane were shown to be protected from exposure to hydrogen peroxide and non-excitotoxic glutamate toxicity modelling oxidative stress (111). Finally, an *in vitro* study of cultured cortical astrocytes exposed to hypoxia and glucose deprivation as experienced following ischaemia/reperfusion injury, showed sulforaphane improved astrocyte cell viability (112).

Nguyen et al. (113) exposed rats to lipopolysaccharide (LPS) inducing maternal and fetal inflammation with consequent growth restriction and developmental delays, which was mitigated following maternal broccoli sprout administration with improvement in birthweight and offspring neurodevelopmental outcomes. A recent large animal model of hypoxic brain injury using piglets was able to show systemic administration of sulforaphane 15-min after hypoxic brain injury was able to improve neuronal survival in the putamen and sensorimotor cortex further highlighting a potential role in preventing neurodevelopmental disorders in the perinatal stroke setting (114).

The therapeutic potential of sulforaphane has been or is currently being investigated in several clinical trials, including for prostate cancer (115), children with autism spectrum disorder (116), subarachnoid haemorrhage (117), preeclampsia (103), and depression (118). Ultimately, sulforaphane is a potent antioxidant regulating phase II detoxification enzyme, which may help to mitigate the pathological processes underpinning disorders of neurodevelopment due to oxidative stress and inflammation. With an excellent safety profile based on animal studies and numerous potentially beneficial effects, sulforaphane represents a potentially powerful fetal therapeutic in pregnancies complicated by impaired placental function, such as preeclampsia and fetal growth restriction (FGR).

### Creatine

Creatine (N-[aminoiminomethyl]-N-methyl glycine) is a nitrogenous amino acid derivative important for cellular energetics as the substrate for the creatine kinase circuit (119, 120). This phosphagen shuttle is integrated into oxidation phosphorylation *via* the mitochondria, as well as glycolysis, with the primary functions of the circuit being immediate temporal energy buffering, i.e., maintaining ATP turnover and the intracellular ATP/ADP ratio; and as a spatial energy buffer to transport high energy phosphagens from sites of ATP production to sites of ATP utilisation (119). The creatine phosphagen system produces ATP more rapidly than any other metabolic system, with the interplay between the different components of the creatine kinase circuit being essential to sustain the bioenergetic demand of a cell (120, 121).

Creatine can be acquired from a diet of meat, fish, and dairy products or synthesised by the body *de novo* (119). In the 1970s, athletes first used dietary creatine supplements as a potential ergogenic aid due to their capacity to maintain cellular energy turnover in tissues with high and fluctuating energy demands (122). Over the years, the pleiotropic effects of creatine on cellular bioenergetics have seen dietary creatine supplements gain attention as a protective therapeutic agent for disorders associated with chronic or acute energy depletion (123–128).

The fetal brain appears to rely on creatine for normal growth and development, with creatine metabolism integral to energy homeostasis during central nervous system development in the embryo, particularly in the growth of dendrites and axons and migration of neuronal growth cones (129, 130). The importance of creatine for adequate brain maturation and function in the neonatal period, and beyond, is highlighted by those infants born with inherited creatine deficiency syndromes (CDS) who often present with progressive neurological deficits, including impaired psychomotor function and seizures (131, 132). Notably, the simple introduction of dietary creatine supplements is often sufficient to restore cerebral creatine and when diagnosed early and managed, those with CDS can live relatively symptom-free. However, much still needs to be done to improve awareness of CDS in the wider community and promote early screening of at-risk infants (133).

In addition to the use of dietary creatine supplements to correct deficiencies, the capacity of creatine to maintain ATP turnover, acid-base balance, and mitochondrial function, along with its antioxidant, vasodilator, and anti-excitotoxic properties, make creatine a candidate for the treatment of ischemic-reperfusion brain injuries (125). Indeed, a 2021 systematic review by Tran et al. (134) of pre-clinical (small animal and rodent) studies assessing creatine supplementation to protect the perinatal brain from hypoxic-ischemic encephalopathy concluded that creatine supplementation during pregnancy showed promise as a prophylactic therapy. Further studies in a large translational sheep model of fetal hypoxia-ischemia have detailed the benefits of high creatine concentration in the fetal circulation before hypoxia on reducing systemic hypoxaemia and interstitial cerebral pyruvate, lactate and reactive oxygen species accumulation in the 72 h after oxygen deprivation (135–137). Investigations into the histopathological and functional consequences of these improvements are underway. Still, based on these studies, it appears a clinical trial of maternal dietary creatine supplementation to reduce the incidence of hypoxic-ischemic perinatal brain injury is imminent. Investigations into the benefits of creatine supplementation during pregnancy and in the early postnatal period following preterm birth are also underway (138).

## Conclusion

Maternal nutrients are essential to supporting a healthy environment for fetal growth and development, particularly neurodevelopment. The maternal diet can be insufficient to provide the necessary micro- and macro-nutrients, with dietary supplementation often recommended in pregnancy. Here we have highlighted several essential nutrients; folate, iodine, vitamin B12, iron, vitamin D, PUFAs, and choline, that play a role in successful fetal neurodevelopment. However, while many of these nutrients require additional supplementation in pregnancy, there is also evidence that excessive supplementation may also be associated with adverse neurodevelopment. Ongoing research to inform who most benefits from supplementation, for how long they should receive additional supplementation and to achieve what target level in the mother for optimal pregnancy outcomes and offspring neurodevelopment is needed.

Beyond supporting normal human development, nutritional supplements in the form of nutraceuticals also hold promise to protect fetal neurodevelopment in the face of pregnancy complications. Sulforaphane, a potent anti-inflammatory and antioxidant, may have the potential to mitigate some of the pathological processes seen in disorders of pregnancy that are associated with inflammation and oxidative stress. Similarly, creatine is integral for cellular bioenergetics and central nervous system development, offering promise of fetal neuroprotection from perinatal asphyxia. Indeed, sulforaphane and creatine have potential benefits to minimise adverse neurodevelopmental outcomes and neuropsychiatric disorders later in life.

Maternal nutrition is essential to fetal neurodevelopment and while this review has outlined what is currently known it is an exciting area to watch, as the complete role of micro- and macro-nutrients on the fetal brain are still far from fully understood.

## Author contributions

SH wrote the Introduction/Conclusion, sections on folate/iodine/vit D/vit B12/iron. NF wrote the section on sulforaphane and the introduction to nutraceuticals, contributed to the overall manuscript structure, and coordinated the referencing. SE wrote the section on creatine and provided input to the remainder of the manuscript. MF provided insight and feedback regarding paediatric neurology. KP coordinated the manuscript and was the main editor. All authors contributed to the article and approved the submitted version.
